# FBXL16 promotes cell growth and drug resistance in lung adenocarcinomas with *KRAS* mutation by stabilizing IRS1 and upregulating IRS1/AKT signaling

**DOI:** 10.1002/1878-0261.13554

**Published:** 2024-01-17

**Authors:** Marion Morel, Weiwen Long

**Affiliations:** ^1^ Department of Biochemistry and Molecular Biology, Boonshoft School of Medicine Wright State University Dayton OH USA

**Keywords:** AKT, FBXL16, IRS1, KRAS, lung adenocarcinoma, protein stability

## Abstract

Lung cancer is the leading cause of cancer‐related deaths worldwide. Lung adenocarcinomas (LUADs) are a major subtype of non‐small‐cell lung cancers (NSCLCs). About 25% of LUADs harbor GTPase *KRAS* mutations associated with poor prognosis and limited treatment options. While encouraging tumor response to novel covalent inhibitors specifically targeting KRASG12C has been shown in the clinic, either intrinsic resistance exists or acquired therapeutic resistance arises upon treatment. There is an unmet need to identify new therapeutic targets for treating LUADs with activating *KRAS* mutations, particularly those with resistance to KRASG12C inhibitor(s). In this study, we have revealed that F‐box/LRR‐repeat protein 16 (FBXL16) is selectively upregulated in LUAD with *KRAS* mutations. It promotes LUAD cell growth and transforms lung epithelial cells. Importantly, *FBXL16* depletion greatly enhances sensitivity to the KRASG12C inhibitor (sotorasib) in resistant cells by downregulating phosphatidylinositol 3‐kinase (PI3K)/protein kinase B (PKB; also known as AKT) signaling. Mechanistically, FBXL16 upregulates insulin receptor substrate 1 (IRS1) protein stability, leading to an increase of IGF1/AKT signaling, thereby promoting cell growth and migration. Taken together, our study highlights the potential of FBXL16 as a therapeutic target for treating LUAD with *KRAS* activating mutations.

## Graphical abstract

In lung adenocarcinomas (LUADs) with *KRAS* activating mutations (*KRAS*‐mut LUADs), the expression level of F‐box/LRR‐repeat protein 16 (FBXL16) is selectively upregulated, leading to the protein stabilization and elevated expression of insulin receptor substrate 1 (IRS1). Thus, the IGF1/IRS1/AKT/S6K signaling is increased, thereby promoting LUAD cell growth, migration, and drug resistance.

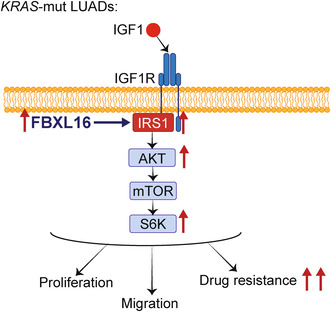

**Abbreviations**
CHX: cycloheximide
CUL1: cullin1
ERK: extracellular signal regulated kinase
IGF1: insulin growth factor 1
IRS1: insulin receptor substrate 1
KRAS: Kirsten rat sarcoma
LRR: leucine‐rich repeat
LUAD: lung adenocarcinoma
LUSC: lung squamous cell carcinoma
NSCLC: non‐small‐cell lung cancers
OS: overall survival
PI3K: phosphoinositide 3‐kinases
S6K: ribosomal protein S6 kinase
SKP1: S‐phase kinase associated protein 1
SRC3: steroid receptor coactivator 3
TMA: tissue microarray

## 1 Introduction

With 1.8 million deaths in 2020, lung cancer is the leading cause of cancer death worldwide [1]. Accounting for 85% of the cases, non‐small‐cell lung cancer (NSCLC) is the most common type and is categorized into different subtypes, including lung squamous cell carcinoma (LUSC) and lung adenocarcinoma (LUAD). LUAD is the most prevalent subtype (around 40%) of NSCLCs and is commonly associated with genetic mutations. Mutation in the Kirsten rat sarcoma (*KRAS*) gene occurs in about 25% of LUAD cases and is implicated in tumor aggressiveness and poor prognosis [2, 3]. Recently, novel covalent inhibitors specifically targeting KRASG12C, such as sotorasib (AMG‐510) and adagrasib (MRTX‐849), have demonstrated encouraging clinical response. However, rapid development of either intrinsic or acquired resistance limits its long‐term efficacy [4, 5, 6, 7, 8]. Thus, it becomes crucial to identify new therapeutic targets and elucidate the underlying mechanisms associated with resistance to improve treatment outcomes.

FBXL16 is an F‐box protein harboring an N‐terminal proline‐rich domain, an F‐box motif, and a C‐terminal leucine‐rich repeat (LRR) domain. Through their F‐box domains, F‐box proteins usually form an SCF (SKP1‐CUL1‐F‐box) complex to constitute an active E3 ubiquitin ligase in which the F‐box protein recruits substrates for protein ubiquitination [9, 10]. While FBXL16 did not show detectable interaction with CUL1 and may not form an active E3 ligase [11, 12], recent studies have demonstrated that FBXL16 is involved in regulating the ubiquitination and stability of oncoproteins, such as MYC, SRC3, CYCLIN D1, and HIF1 alpha [13, 14, 15, 16]. By upregulating MYC stability, FBXL16 promotes cancer cell growth and migration [13]. In breast cancer, FBXL16 upregulates SRC3, which leads to an increase in cell proliferation through the activation of AKT pathway [15]. While these previous studies have identified FBXL16 as a potential candidate implicated in oncogenesis, its specific function in NSCLC, and particularly in LUAD, remains to be explored.

In this study, we have found that FBXL16 is overexpressed in LUADs, and its high expression level is positively associated with poor prognosis. Of important note, FBXL16 expression level is even significantly higher in LUADs with *KRAS* mutations than in LUADs with wild type *KRAS*. In line with these clinical findings, FBXL16 promotes LUAD cell growth and transforms lung epithelial cells. In addition, we found that depletion of FBXL16 in LUAD cells with resistance to the KRASG12C inhibitor, sotorasib, greatly enhanced their sensitivity through the downregulation of PI3K/AKT signaling. Mechanistically, FBXL16 upregulates IRS1 protein stability, leading to increase of IGF1/AKT signaling and promotion of cell growth and migration. Taken together, our study has identified an important role for FBXL16 in promoting growth and drug resistance in LUADs with *KRAS* mutations and revealed FBXL16 as a potential target for treating LUADs with *KRAS* activating mutations.

## 2 Materials and methods

### 2.1 Cell culture and chemicals

Lung cancer cell lines [H520 (RRID:CVCL_1566), H2170 (RRID:CVCL_1535), H1703 (RRID:CVCL_1490), H1299 (RRID:CVCL_0060), H1395 (RRID:CVCL_1467), H1437 (RRID:CVCL_1472), SW900 (RRID:CVCL_1731), Calu‐1 (RRID:CVCL_0608), H23 (RRID:CVCL_1547), H441 (RRID:CVCL_1561), A549 (RRID:CVCL_0023), H358 (RRID:CVCL_1559), SW1573 (RRID:CVCL_1720) and H1792 (RRID:CVCL_1495)] were obtained from ATCC (American Type Culture Collection) and maintained in RPMI1640 medium (Gibco 22400‐089, Grand Island, NY, USA). HEK293T (RRID:CVCL_0063) and HeLa (RRID:CVCL_0030) cell lines were obtained from ATCC and maintained in DMEM (Gibco 11965‐092). Both RPMI and DMEM media were supplemented with 10% FBS (Gibco 26140‐095) and 1% penicillin/streptomycin (Gibco 15070‐063). NL‐20 (RRID:CVCL_3756) cell line was obtained from ATCC and maintained in Ham's F‐12K (Kaighn's) medium (Gibco 21127‐022) supplemented with 4% FBS, 1% penicillin/streptomycin, 1× MEM Non‐Essential Amino Acids (Gibco 11140‐050), 1× Transferrin/Insulin (Gibco 41400‐045), 500 ng·mL^−1^ Hydrocortisone (Sigma H0888, Burlington, MA, USA) and 10 ng·mL^−1^ hEGF (Peprotech AF‐100‐15, Cranbury, NJ, USA). BEAS‐2B (RRID:CVCL_0168) cell line was obtained from ATCC and maintained in BEBM™ Bronchial Epithelial Cell Growth Basal Medium (Lonza CC‐3171, Basel, Switzerland) supplemented with BEGM™ Bronchial Epithelial SingleQuots™ Kit (Lonza CC‐4175). All cell lines were originally authenticated by ATCC. All experiments were performed with mycoplasma‐free cells. Sotorasib (AMG510) was purchased from MedChemExpress (HY‐114277) and was reconstituted in DMSO.

### 2.2 Transient siRNA and plasmid transfections

All FBXL16‐expressing plasmids used in this study were previously described [13]. AllStars Negative Control siRNA (Qiagen SI03650318, Germantown, MD, USA), ON‐TARGETplus Nontargeting siRNA Control Pool (Horizon D‐001810‐10, Cambridge, UK) and Silencer^®^ Select Negative Control siRNA (Ambion 4390843, Austin, TX, USA) were used as non‐silencing controls. Hs_FBXL16_7 FlexiTube siRNA (Qiagen SI04277203), Hs_FBXL16_8 FlexiTube siRNA (Qiagen SI04287276), or ON‐TARGETplus Human FBXL16 siRNA (Horizon L‐016797‐00) was used to silence FBXL16. Silencer^®^ Select KRAS siRNA (Ambion 4390824) was used to target KRAS. Hs_FBXW8_2 FlexiTube siRNA (Qiagen SI00156030) was used to target FBXW8. The following siRNA sequence 5′‐AAGUGGAAUUUGUGGAACAUCdTdT‐3′ was used to target β‐TRCP1/2. For gene knockdown, cells were transfected with 25 nm of siRNA using Dharmafect 1 (Dharmacon T‐2001, Lafayette, CO, USA) following manufacturer instructions. Transient plasmid transfections were performed using FuGene HD (Promega, Madison, WI, USA) or Lipofectamine 3000 (Invitrogen, Carlsbad, CA, USA) following manufacturer instructions.

### 2.3 Generation of stable cells

The retroviral expressing plasmid MSCV‐N‐Flag‐HA‐GFP was a gift from Wade Harper (Addgene plasmid # 41034, Watertown, MA, USA; http://n2t.net/addgene:41034; RRID: Addgene_41 034) [17]. Retroviral FLAG‐tagged FBXL16 constructs were generated by digesting pSG5‐FLAGFBXL16, pSG5‐FLAGFBXL16ΔFbox and pSG5‐FLAGFBXL16ΔLRR [13] with HindIII and EcoRI restriction enzymes and the insertion of the digested FLAGFBXL16 fragments into the MSCV retroviral plasmid between BglII and EcoRI restriction sites.

Retroviral particles were produced in HEK293T cells by cotransfecting the retroviral expression construct with retroviral packaging plasmids (gag/pol and VSV‐G, both were kindly provided by Wade Harper at Harvard Medical School [17]). The retroviral particles were harvested 48 h after transfection and concentrated using RetroX concentrator (Takara, 631455, San Jose, CA, USA) following manufacturer's instructions. NL‐20 and BEAS‐2B cell lines with stable overexpression of GFP, FBXL16, FBXL16ΔFbox or FBXL16ΔLRR were generated by transducing cells with each corresponding retrovirus in the presence of 1 μg·mL^−1^ Polybrene. 48 h after transduction, cells were selected by puromycin (1–2 μg·mL^−1^) for a minimum of 10 days until reaching a stable cell growth rate. The stable cell lines were validated by Western blotting and then used for other experiments.

### 2.4 Western blot analysis

Cells were lysed with EBC buffer (50 mm Tris, pH 7.5, 150 mm NaCl, 0.5% Nonidet P‐40) supplemented with 1× cOmplete™ Protease Inhibitors (Roche, Basel, Switzerland) and 1× Phosphatase Inhibitor Cocktail (Sigma‐Aldrich P0044, Burlington, MA, USA). Western blotting was performed by SDS/PAGE followed by transfer of the proteins onto nitrocellulose membranes and blocking the membranes with 5% nonfat milk in TBS containing 0.01% Tween‐20 following the procedures previously described [18]. The following antibodies were used: anti‐FBXL16 (GeneTex, GTX31424), anti–β‐actin (Sigma‐Aldrich, A5316), anti‐GAPDH (Cell Signaling Technologies, Danvers, MA, USA, 2118), anti‐IRS1 (Sigma‐Aldrich 06‐248), anti–KRAS (Santa‐Cruz Biotechnology, Dallas, TX, USA, sc‐30), anti‐HA (Sigma‐Aldrich, H3663), anti‐FLAG (Sigma‐Aldrich, F1804), anti‐phospho‐AKT (Cell Signaling Technologies, 4060), anti‐AKT1 (Cell Signaling Technologies, 2967), anti‐phospho‐p70S6K (Cell Signaling Technologies, 9205), anti‐p70S6K (Cell Signaling Technologies, 2708), anti‐phospho‐S6 (Cell Signaling Technologies, 5364), anti‐S6 (Cell Signaling Technologies, 2217), anti‐phospho‐ERK1/2 (Cell Signaling Technologies, 4370), anti‐ERK1/2 (Cell Signaling Technologies, 9102), anti–mouse‐HRP (Bio‐Rad, Hercules, CA, USA, 170‐6516), and anti–rabbit‐HRP (Bio‐Rad, 170‐6515). The band intensity (the relative level) was quantified and normalized to the loading control (GAPDH or Actin) using imagej software (NIH, Bethesda, MD, USA).

### 2.5 Immunohistochemistry

Tissue microarrays (TMA) were purchased from US Biomax (LC10012a) and Yale Pathology Tissue Services (YPTS, TMA #310). After removal of paraffin and rehydration, tissues were treated with citrate‐based antigen unmasking solution (Vector Laboratories, H3300, Newark, CA, USA) following manufacturer instructions. After permeabilization with 0.3% Triton X‐100, tissues were blocked with 10% Normal Goat Serum (NGS, Vector Laboratories S‐1000), followed by incubation with primary antibodies [anti‐FBXL16 rabbit IgG, 1 : 600 dilution; anti‐IRS1 rabbit IgG, 1 : 250 dilution; or normal rabbit IgG (negative control), 1.7 μg·mL^−1^] overnight at 4 °C. After washing, TMA were incubated with goat anti‐rabbit IgG antibodies, biotinylated (1 : 200, Vector Laboratories, BA‐1000). Signal was enhanced using Vectastain^®^ Elite^®^ ABC‐HRP kit (Vector Laboratories PK‐6200) following manufacturer's instructions, and stained using 3,3′‐Diaminobenzidine (DAB, Acros Organics 11090500, Geel, Belgium). Finally, tissues were counterstained with Hematoxylin (Ricca 3530‐32, Arlington, TX, USA) and imaged using EVOS XL Core microscope. Staining of the sections labeled with normal rabbit IgG was considered background. The protein staining intensity of each specimen was graded on a semiquantitative scale (0: indicates no staining relative to background, 1: weak, 2: moderate, 3: intense).

### 2.6 Protein stability assay

As described previously [13], a stock solution (10 mg·mL^−1^) of cycloheximide (CHX) (Sigma‐Aldrich, C7698) was prepared in sterile water. 30 h after transient transfection, cells were treated with 100 μg·mL^−1^ CHX for different time periods as indicated in the figures. After Western blot analysis, IRS1 protein level at each time point was normalized to that of β‐actin or GAPDH, and the normalized IRS1 protein level at 0 min time point was arbitrarily set as 1. The protein half‐life was calculated with graphpad prism 9 software (Graphpad Software, Inc., La Jolla, CA, USA) using the one‐phase exponential decay model.

### 2.7 Immunoprecipitation

Cell lysates were precleared by incubation with EZView™ Red Protein A Affinity gel (Sigma‐Aldrich, P6486). IRS1 antibody was then added to the precleared cell lysates, and the mixture was incubated overnight. Next, the mixture was incubated with EZView™ Red Protein A Affinity gel. After 1 h, beads were washed three times with lysis buffer. The immunoprecipitated proteins were then eluted off the beads with 2× Laemmli sample buffer, followed by Western blot analysis as previously described [13].

### 2.8 Two‐chamber Transwell cell migration assay

Cell migration was analyzed by using a modified two‐chamber Transwell system (BD Biosciences) following the manufacturer's instructions. The bottom well was filled with medium containing 0.5% fetal bovine serum. After serum starvation for 6 h, cells were trypsinized, washed, and resuspended in serum‐free medium. Resuspended cells were then added into each Transwell insert in presence of 0.1% BSA (vehicle control) or 10 nm IGF1 (Peprotech 100‐11) and were allowed to migrate in a 37 °C cell incubator for 18 h. Next, as previously described in [13], cells on the upper surface of the insert membrane were removed with cotton swabs. The migrated cells attached to the undersurface of the insert membrane were then fixed in 4% paraformaldehyde for 15 min and stained with 0.5% crystal violet solution for 10 min. Migrated cells were assessed using an EVOS XL Core microscope and quantified using imagej.

### 2.9 Proliferation assays

Cell proliferation was determined using FluoReporter™ Blue Fluorometric dsDNA Quantitation Kit (Invitrogen, F2962) following the manufacturer's instructions.

### 2.10 Soft agar colony formation assay

Anchorage‐independent colony formation assay was performed as previously described [13]. Cells were grown in 0.4% (NL20) or 0.35% (BEAS2B) agarose in a 37 °C humidified cell culture incubator. Fresh complete medium was added to replace the old medium every 2–3 days for a total of 21 days. Cells were stained with 1 mg·mL^−1^ of Nitro blue tetrazolium chloride (NBT, Electron Microscopy Sciences 19035‐10, Hatfield, PA, USA) overnight in a 37 °C humidified cell culture incubator. Cell colony formation was quantified using imagej software.

### 2.11 Dataset analysis

GEPIA2 web server [19] was used to compare the mRNA levels of *FBXL16* in LUAD patient samples (TCGA dataset) and lung normal tissue samples (TCGA + GTEx datasets). Survival analysis was performed using Kaplan‐Meier web server [20] using all available datasets combined (GSE102287, GSE14814, GSE157011, GSE19188, GSE29013, GSE30219, GSE31210, GSE3141, GSE31908, GSE37745, GSE43580, GSE4573, GSE50081, GSE68465, GSE77803, GSE8894 and TCGA). IRS1 protein expression in LUAD patient samples were obtained from Clinical Proteomic Tumor Analysis Consortium (CPTAC) dataset [21] and analyzed using cBioPortal web server [22, 23].

### 2.12 Statistics

Data are expressed as mean ± SEM. All experiments were repeated at least three times and a representative figure is presented. Statistical significance was determined by Welsh's *t* test, one‐way analysis of variance (ANOVA), or two‐way ANOVA, as indicated in each figure legend, and a *P* value of < 0.05 was considered statistically significant (*P < 0.05; **P < 0.01; ***P < 0.001).

## 3 Results

### 3.1 FBXL16 expression is upregulated in LUADs with *KRAS* mutations

**Fig. 1:**
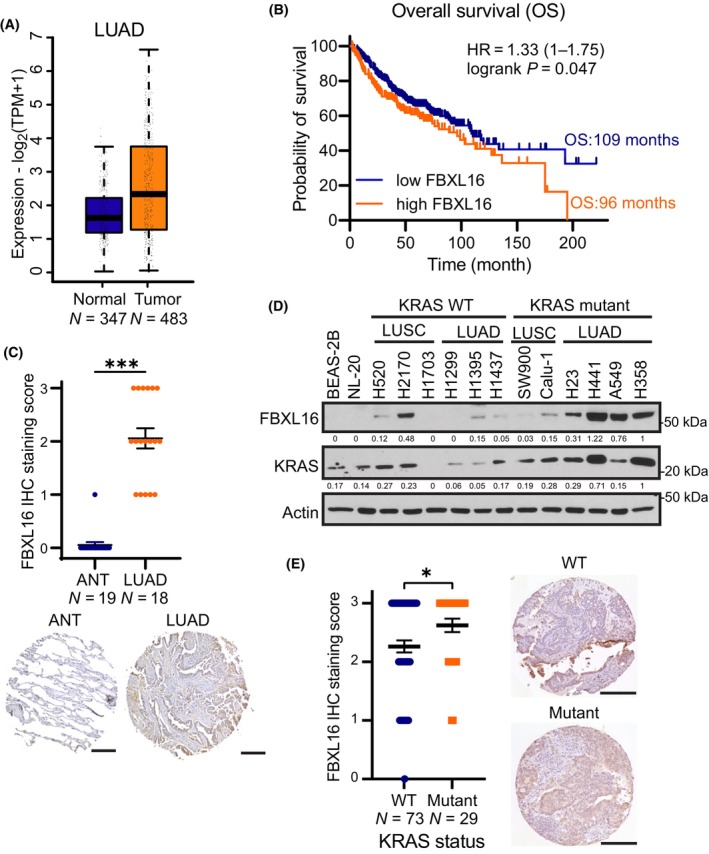
FBXL16 is overexpressed in lung adenocarcinomas with *KRAS* mutations. (A) Expression of FBXL16 in normal tissues (*N* = 347) versus LUADs (*N* = 483). This analysis was performed on TCGA (tumor and normal tissue samples) and GTEx (normal tissue samples) datasets using GEPIA2 web server [19]. (B) LUAD patients were split into two groups as lower tercile (T1, low FBXL16, *N* = 342, median overall survival: 109 months) versus upper tercile (T3, high FBXL16, *N* = 178, median overall survival: 96 months) using Kaplan–Meier plotter web server [20]. (C) IHC analysis of FBXL16 protein in a TMA constituted of adjacent normal tissues (ANT, *N* = 19) and lung adenocarcinoma (LUAD, *N* = 18) samples. Pictures of representative cores are shown below (scale bar: 250 μm). (D) Western blot analysis of FBXL16 and KRAS protein levels in two immortalized (non‐cancerous) lung epithelial cell lines (BEAS‐2B and NL‐20) and NSCLC (LUSC or LUAD) cell lines harboring either wild‐type (WT) *KRAS* or *KRAS* mutants (*n* = 3). (E) IHC analysis of FBXL16 protein in a TMA constituted of LUAD samples harboring *KRAS* WT (*N* = 73) or *KRAS* mutations (*N* = 29). Pictures of representative cores are shown on the side (scale bar: 250 μm). Values in the graphs (C, E) represent mean ± SEM and statistical significance was determined by Welsh's *t*‐test (*P < 0.05; ***P < 0.001).

While previous studies have identified a role for FBXL16 in breast cancer [14, 15] and its potential implication in lung cancer [13], the function of FBXL16 in NSCLCs remains to be elucidated. By data mining, we found that *FBXL16* is highly expressed at mRNA level in lung adenocarcinomas (LUADs) (Fig. 1A). In addition, our analysis revealed a notable correlation between high *FBXL16* mRNA expression and a decrease in overall survival (OS) in patients diagnosed with LUADs (Fig. 1B). To validate these findings, we analyzed FBXL16 protein expression level by immunohistochemistry (IHC) on a tissue microarray (TMA) containing LUAD samples (*N* = 18) and adjacent normal tissue samples (ANT, *N* = 19). Indeed, FBXL16 protein is significantly overexpressed in LUADs, while low or negligible expression of FBXL16 was detected in adjacent normal tissues (Fig. 1C). The specificity of FBXL16 antibody was validated by incubating the same TMA with normal rabbit IgG, which showed virtually negative staining (Fig. S1). Next, we analyzed FBXL16 protein expression in a panel of different NSCLC cell lines as well as two immortalized lung epithelial cells. Interestingly, we observed that FBXL16 protein level is selectively upregulated in LUADs carrying activating *KRAS* mutations and is undetectable in normal epithelial cells BEAS‐2B and NL‐20 (Fig. 1D). This prompted us to investigate whether FBXL16 is differentially upregulated in LUADs expressing KRAS mutations versus those expressing wild‐type KRAS. We then examined FBXL16 protein levels by IHC in a TMA consisting of LUAD samples with known *KRAS* mutation status. Indeed, we observed that FBXL16 protein expression level is significantly higher in LUADs with *KRAS* mutations than LUADs with wild‐type *KRAS* (Fig. 1E). Altogether, these results demonstrate that FBXL16 is upregulated in LUADs, particularly in those with *KRAS* mutations, and its elevated expression is associated with decrease in overall survival.

### 3.2 FBXL16 promotes LUAD cell growth and transforms lung epithelial cells

**Fig. 2:**
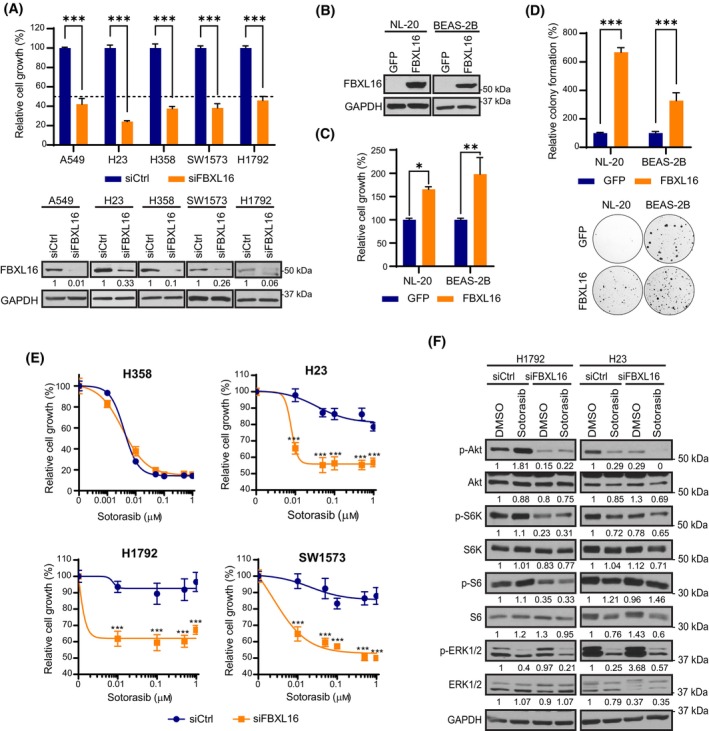
FBXL16 promotes lung cell growth and resistance to sotorasib. (A) LUAD cell lines A549 (*KRASG12S*) (*n* = 3), H23 (*KRASG12C*) (*n* = 3), H358 (*KRASG12C*) (*n* = 3), SW1573 (*KRASG12C*) (*n* = 4) and H1792 (*KRASG12C*) (*n* = 3) were transiently transfected with a negative control siRNA (siCtrl) or a siRNA targeting *FBXL16* (siFBXL16). 5 days post‐transfection, cell growth (proliferation) was determined by dsDNA content measurement. Cell growth relative to siCtrl was shown in bar graphs. The knockdown of FBXL16 was confirmed by Western blot analysis. (B) Western blot analysis of NL‐20 and BEAS‐2B cell lines with stable overexpression of GFP or FBXL16. (C) Cell growth of NL‐20 (*n* = 3) and BEAS‐2B (*n* = 3) cells with stable overexpression of GFP or FBXL16 was determined by measuring the dsDNA content after 5 days of growth. Cell growth relative to GFP control was shown in bar graphs. (D) Stable NL‐20 and BEAS‐2B cell lines with overexpression of GFP or FBXL16 were grown in soft agar. After 21 days, colonies were stained and quantified using imagej software. Values are normalized to the GFP control and representative wells are shown below (*n* = 5). (E) LUAD cell lines harboring *KRASG12C*, including H358, H23, H1792 and SW1573, were transiently transfected with siCtrl or siFBXL16 and treated with sotorasib for 72 h. dsDNA content was measured, and values were normalized to each DMSO control (0 μm) which was set to 100%. Non‐linear regression curves were added using prism 9 software, and statistical significance was determined by two‐way ANOVA (*n* = 3). (F) Western blot analysis of H1792 and H23 cell lines which were transfected with siCtrl or siFBXL16 and then treated with DMSO or 1 μm sotorasib for 24 h. In (A), (C), (D), and (E), values in graph represent mean ± SEM and statistical significance was determined by two‐way ANOVA (*P < 0.05; **P < 0.01; ***P < 0.001).

We then investigated the role of FBXL16 on LUAD cell growth. First, we depleted FBXL16 in several LUAD cell lines harboring *KRAS* mutations and determined the effect of FBXL16 knockdown on cell growth. As shown in Fig. 2A and Fig. S2, knockdown of FBXL16 led to a drastic decrease in cell proliferation in all tested cell lines. To confirm cell proliferation‐promoting role of FBXL16, we generated cell lines with stable overexpression of FBXL16 using the immortalized lung epithelial cell lines BEAS‐2B and NL‐20 wherein no endogenous FBXL16 protein was detected as shown in Fig. 1D. We then compared their growth with control cell lines stably expressing GFP (Fig. 2B). Notably, overexpression of FBXL16 led to a significant increase in cell proliferation under both 2‐dimension (2D) condition (Fig. 2C) and anchorage‐independent colony formation in soft agar (*in vitro* 3D transformation assay) when compared to GFP control cell lines (Fig. 2D). Altogether, these findings strongly indicate that FBXL16 promotes LUAD cell growth and stimulates transformation of lung epithelial cells.

### 3.3 FBXL16 promotes resistance to KRASG12C inhibitor by upregulating PI3K/AKT pathway

While KRAS was historically described as “undruggable”, novel covalent inhibitors specifically targeting KRASG12C, such as sotorasib (AMG‐510), were recently approved for the treatment of NSCLC patients harboring KRASG12C mutation [7, 24, 25, 26]. Unfortunately, intrinsic and acquired resistance to KRASG12C inhibitors has already been extensively documented [4, 27, 28, 29]. Given the significant upregulation of FBXL16 in LUADs with *KRAS* mutations, we hypothesized that FBXL16 might contribute to resistance to KRASG12C inhibitors. Previous investigations have revealed varying degrees of sensitivity to the inhibitor among different cell lines: high sensitivity (e.g., H358), partial resistance (e.g., H23), or complete resistance (e.g., H1792 and SW1573) [30]. Hence, we assessed the impact of depleting FBXL16 in these different cell lines on their response to sotorasib. Interestingly, while FBXL16 knockdown did not influence the response of the highly sensitive cell line H358, it remarkably enhanced the sensitivity of H23, H1792, and SW1573 cell lines to sotorasib (Fig. 2E).

Next, we aimed to elucidate the mechanism through which FBXL16 affects the response of LUAD cells to KRASG12C inhibitor. By Western blot analysis, we discovered that the depletion of FBXL16 reduced the phosphorylations of AKT and S6K (Fig. 2F, Fig. S3). Notably, in highly resistant H1792 cells, while treatment with sotorasib alone resulted in an increase in phosphorylation levels of AKT, S6K, and S6 as previously reported [30], the combination of siFBXL16 and sotorasib treatment abolished this upregulation and actually even reduced the phosphorylation levels compared to siCtrl/DMSO condition (Fig. 2F). Upregulation of PI3K/AKT pathway has been known as a major mechanism of resistance to KRASG12C inhibition [30, 31, 32, 33]. Therefore, we speculate that the depletion of FBXL16 enhanced the sensitivity of resistant cells to sotorasib via downregulating the PI3K/AKT signaling.

### 3.4 FBXL16 protein level positively correlates with IRS1 protein level in LUADs with KRAS mutations

**Fig. 3:**
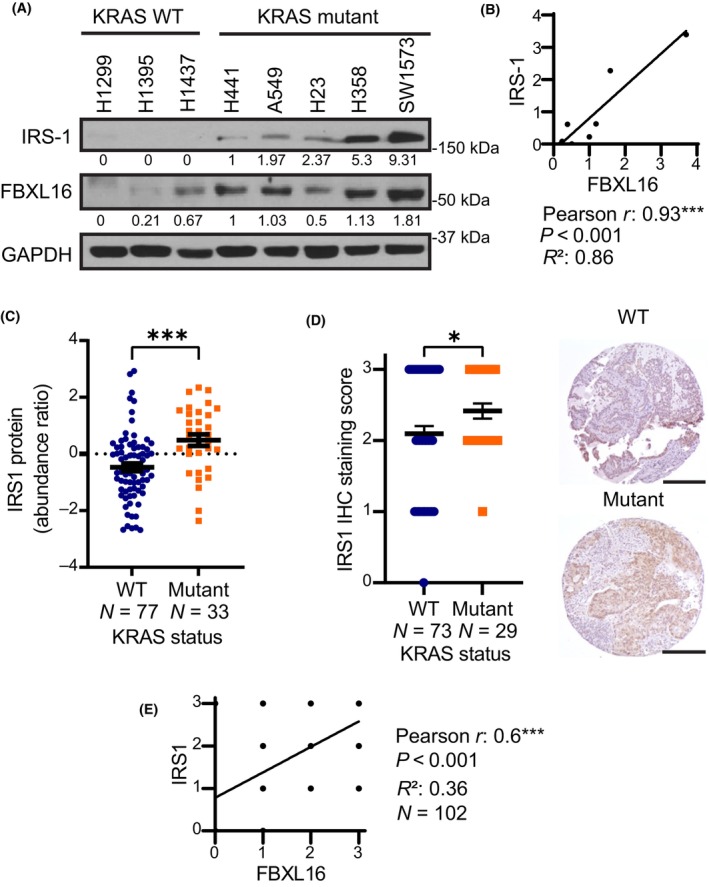
IRS1 protein expression is upregulated and positively correlates with FBXL16 in LUADs with *KRAS* mutations. (A) Western blot analysis of FBXL16 and IRS1 protein levels in a panel of LUAD cell lines (*n* = 3). (B) Protein band intensity (expression level) was quantified using imagej software, normalized (to that of GAPDH), and plotted to determine the correlation between FBXL16 and IRS1 in LUAD cell lines through Pearson‐correlation analysis using Prism 9 software (*N* = 8). (C) IRS1 protein expression in LUAD patient samples harboring *KRAS* WT (*N* = 77) or *KRAS* mutant (*N* = 33) using the Clinical Proteomic Tumor Analysis Consortium (CPTAC) dataset [21]. (D) IHC analysis of IRS1 protein expression in the same TMA of LUADs as described in Fig. 1E. Pictures of representative cores are shown on the side (scale bar: 250 μm). (E) Scatter plot showing the correlation between FBXL16 and IRS1 protein expression levels in LUAD patient samples by Pearson‐correlation analysis using Prism 9 software (*N* = 102). In (C) and (D), values in graph represent mean ± SEM and statistical significance was determined by Welsh's *t*‐test (*P < 0.05; ***P < 0.001).

Previous studies have shown the upregulation of IGFR/IRS1/PI3K signaling and its essential role in promoting resistance to KRASG12C inhibitors [30, 31]. In addition, insulin receptor substrates 1 (IRS1) and 2 (IRS2) were shown to be required for KRAS‐mutant induced lung tumorigenesis [34]. Interestingly, in our proteomic mass spectrometry analysis of breast cancer cell line T47D, we observed a significant decrease in IRS1 protein level upon FBXL16 knockdown (data not shown). We wondered whether FBXL16 positively regulates IRS1 protein level in LUADs. First, we found that similar to FBXL16, IRS1 protein level is selectively upregulated in LUAD cell lines with *KRAS* mutation (Fig. 3A). Pearson‐correlation analysis revealed a nearly perfect correlation between IRS1 level and FBXL16 level in LUAD cell lines (*r* = 0.93, *R* ^2^ = 0.86, *P*‐value < 0.001) (Fig. 3B). We then further looked into this by examining IRS1 protein expression in LUAD patient samples. By data mining using the Clinical Proteomic Tumor Analysis Consortium (CPTAC) dataset, we found that IRS1 protein level was significantly upregulated in LUADs with *KRAS* mutation compared to LUADs expressing wild‐type *KRAS* (Fig. 3C). Similarly, IRS1 protein level was significantly higher in LUADs with *KRAS* mutations than LUADs‐wild‐type *KRAS* (Fig. 3D) in the TMA we observed FBXL16 upregulation in LUADs‐*KRAS* mutations shown in Fig. 1E. Also, a Pearson‐correlation analysis of FBXL16 and IRS1 staining in this TMA revealed a positive correlation between these two proteins in LUAD patient samples (*r* = 0.6, *R* ^2^ = 0.36, *P*‐value<0.001) (Fig. 3E). Taken together, these results demonstrate that both FBXL16 and IRS1 are upregulated in LUADs with *KRAS* mutations and there is a significant positive correlation between them, implying a role for FBXL16 in regulating IRS1 signaling.

### 3.5 FBXL16 upregulates IRS1 protein stability

**Fig. 4:**
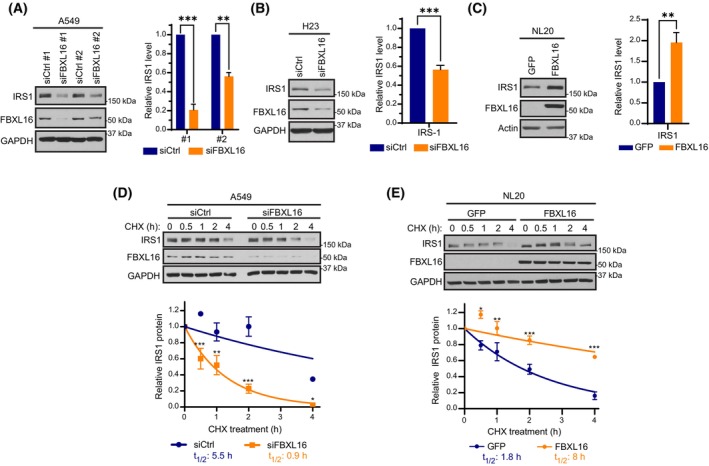
FBXL16 upregulates IRS1 protein stability. (A, B) Western blot analysis of A549 (A) and H23 (B) cells after transient transfection with siCtrl or siFBXL16 (*n* = 3). (C) Western blot analysis of NL‐20 cells with stable overexpression of GFP or FBXL16 (*n* = 3). (D) A549 cells were transiently transfected with siCtrl or siFBXL16. 30 h post transfection, protein translation was inhibited with CHX (100 μg·mL^−1^) for different times as indicated, followed by Western blot analysis. IRS1 protein level at each time point was normalized to that of GAPDH, and the normalized IRS1 protein level at 0‐h time point was set as 1. IRS1 half‐life (t_1/2_) was determined from the exponential curve equation calculated using the one‐phase exponential decay model (prism 9 software) (*n* = 3). (E) NL‐20 cells with stable overexpression of GFP or FBXL16 were treated with CHX for different times and the half‐life of endogenous IRS1 protein was determined as in (D) (*n* = 3). In all experiments, protein band intensity (expression level) was quantified using imagej software and normalized to that of GAPDH or Actin. Values in graphs represent mean ± SEM and statistical significance was determined by one‐way ANOVA (A), t‐test (B, C) and two‐way ANOVA (D, E) (*P < 0.05; **P < 0.01; ***P < 0.001).

We then tested whether FBXL16 upregulates IRS1 protein level in LUADs. First, we determined the effect of FBXL16 depletion on IRS1 in several LUAD cell lines (Fig. 4A,B, Fig. S4A). In all cell lines, knockdown of FBXL16 resulted in a significant reduction of IRS1 protein level. Conversely, overexpression of FBXL16 in both NL‐20 and BEAS‐2B lung epithelial cell line significantly increased endogenous IRS1 protein level (Fig. 4C, Fig. S4A). Moreover, exogenous expression of FBXL16 by cDNA transfection following the knockdown of endogenous FBXL16 rescued the level of IRS1 protein expression (Fig. S4B). In a previous study, we found that FBXL16 upregulates C‐MYC protein level by increasing its protein stability [13]. To explore if FBXL16 affects IRS1 protein level through this mechanism, we investigated the impact of silencing FBXL16 on IRS1 protein stability by treating cells with Cycloheximide (CHX), a protein translation inhibitor. Indeed, knockdown of FBXL16 remarkably decreased IRS1 stability (protein half‐life) (Fig. 4D, Fig. S4C). On the contrary, stable overexpression of FBXL16 in NL‐20 cells stabilized IRS1 protein with a 4.4‐fold increase in half‐life (Fig. 4E) as compared to that in NL‐20‐GFP control cells. These results suggest that FBXL16 plays a crucial role in controlling IRS1 protein stability and expression level in LUADs.

FBXL16 upregulates MYC protein stability by antagonizing the activity of another F‐box protein, FBW7 [13]. IRS1 was shown to be degraded following its ubiquitination by different F‐box proteins, including βTRCP [35] and FBXW8 [36]. We thus tested whether FBXL16 regulates IRS1 through affecting βTRCP or FBXW8. Knockdown of FBXL16 had similar effect on IRS1 with or without the silencing of either βTRCP or FBXW8 (Fig. S5), suggesting that FBXL16 regulates IRS1 independent of βTRCP and FBXW8.

### 3.6 FBXL16 upregulates IGF1/IRS1/AKT signaling and IGF1‐induced cell growth and migration

**Fig. 5:**
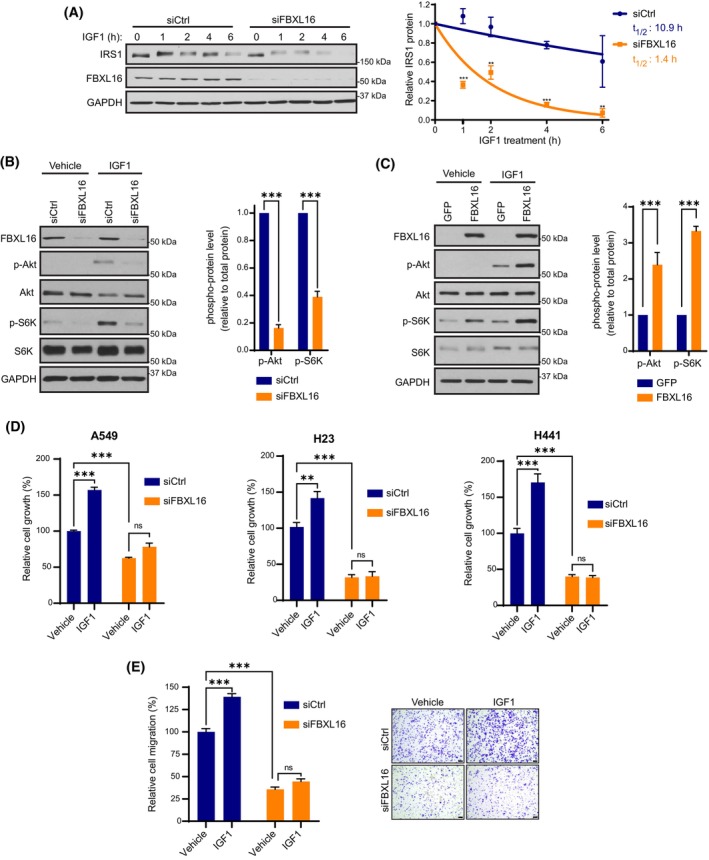
FBXL16 upregulates IGF1/IRS1/AKT signaling. (A) A549 cells were transfected with siFBXL16 or siCtrl. After serum starvation, cells were treated with IGF1 (10 nm) for different times as indicated, followed by Western blot analysis of FBXL16 protein turnover induced by IGF1 stimulation. IRS1 protein level at each time point was normalized to that of GAPDH, and the normalized IRS1 protein level at 0‐h time point was set as 1. IRS1 half‐life (t_1/2_) was determined from the exponential curve equation calculated using the one‐phase exponential decay model (prism 9 software) (*n* = 3) (B) Western blot analysis of phosphorylations of AKT (p‐AKT) and S6K (p‐S6K) in H23 cells transfected with siCtrl or siFBXL16 and then stimulated with IGF1 (10 nm) or vehicle control (0.1% BSA) (*n* = 3). (C) Western blot analysis of phosphorylations of AKT (p‐AKT) and S6K (p‐S6K) in NL‐20 cells with stable overexpression of GFP or FBXL16 stimulated with IGF1 or control vehicle after serum starvation (*n* = 3). (D) A549 (*n* = 3), H23 (*n* = 3) or H441 (*n* = 4) cells were transfected with siCtrl or siFBXL16 and grown in RPMI1640 medium supplemented with 0.5% charcoal‐stripped serum and IGF1 (10 nm) or vehicle control (0.1% BSA). After 5 days, cell growth was measured by dsDNA content measurement. Cell growth relative to the “siCtrl + vehicle” condition (arbitrarily set as 100%) was shown in bar graphs. (E) H23 cells were transfected with siCtrl or siFBXL16. After serum‐starvation for 6 h, their ability to migrate with or without IGF1 stimulation was analyzed by a two‐chamber Transwell assay using medium containing 0.5% FBS in the bottom chamber as chemoattractant. The migration ability of cells is presented as the percentage of the number of migrated cells per field normalized to the “siCtrl + vehicle” condition (*n* = 3). Representative images of migrated cells were shown on the right (Scale bars: 100 μm). In (A–C), protein band intensity (expression level) was quantified using imagej software and normalized to that of GAPDH. Values in graphs represent mean ± SEM and statistical significance was determined by two‐way ANOVA (**P < 0.01; ***P < 0.001).

Following IGF1 stimulation, IRS1 is phosphorylated on tyrosine residues, creating a docking site for PI3K recruitment and subsequent activation of the AKT pathway [37, 38]. Through feedback regulation, IRS1 is then phosphorylated on serine and threonine residues, triggering its degradation [35, 36] and signaling termination. To investigate the potential involvement of FBXL16 in IGF1‐induced IRS1 degradation, we depleted FBXL16 in A549 cells. Following overnight serum‐starvation, cells were stimulated with IGF1 (10 nm). Interestingly, knockdown of FBXL16 greatly accelerated IRS1 degradation upon IGF1 stimulation (7.8‐fold faster) (Fig. 5A), implying that FBXL16 upregulates (sustains) IGF1/IRS1/AKT signaling. In support of this, depletion of FBXL16 significantly reduced phosphorylations of AKT and S6K stimulated by IGF1 (Fig. 5B). Conversely, stable overexpression of FBXL16 significantly increased the phosphorylations of AKT and S6K in NL‐20 upon stimulation with IGF1 (Fig. 5C). In summary, FBXL16 upregulates IGF1‐induced IRS1/AKT signaling.

We then determined whether FBXL16 promotes IGF1‐induced cell growth and migration. A549, H23 or H441 LUAD cell lines were transfected with siFBXL16 or siCtrl. Cells were cultured for 5 days in 0.5% charcoal‐stripped serum (for reducing the concentration of growth factors while maintaining cell viability) and treated daily with IGF1 or a vehicle control. Indeed, depletion of FBXL16 abolished the stimulating effect of IGF1 on cell growth (Fig. 5D) as well as cell migration (Fig. 5E). Taken together, these results show that FBXL16 promotes IGF1/IRS1/AKT signaling and IGF1‐induced cell growth and migration in LUAD cells.

### 3.7 Both F‐box and LRR domains are important for FBXL16 in stabilizing IRS1 and promoting IGF1 signaling

**Fig. 6:**
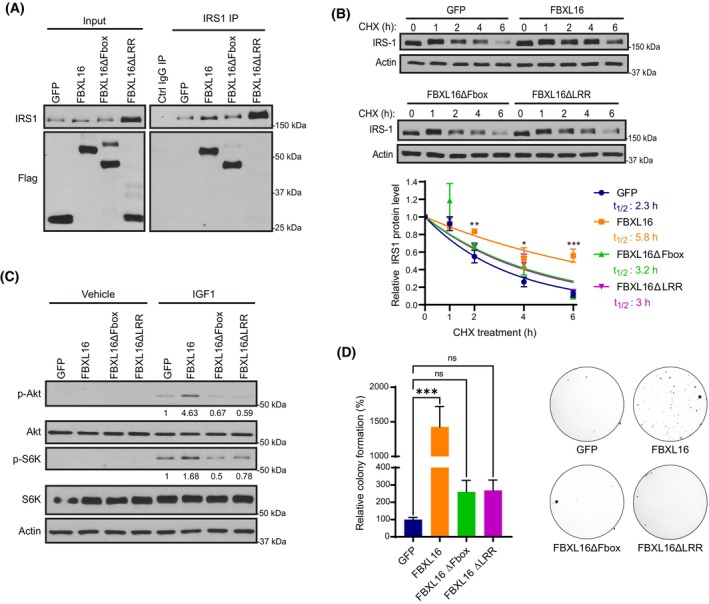
Both F‐box and LRR domains are important for FBXL16 in stabilizing IRS1 and promoting IGF1 signaling. (A) IRS1 protein was immunoprecipitated from NL‐20 cells with stable expression of Flag‐GFP, Flag‐FBXL16, Flag‐FBXL16ΔFbox or Flag‐FBXL16ΔLRR. Immunoprecipitation (IP) using a normal rabbit IgG served as an IP control (Ctrl IgG IP). Western blot analysis was then performed to examine the interactions of IRS1 with the different Flag‐tagged proteins (*n* = 3). (B) NL‐20 with stable overexpression of Flag‐GFP, Flag‐FBXL16, Flag‐FBXL16ΔFbox or Flag‐FBXL16ΔLRR were treated with cycloheximide (CHX, 100 μg·mL^−1^) for different times (hours), followed by Western blot analysis of protein levels. Protein band intensity (expression level) was quantified using imagej software. IRS1 protein level at each time point was normalized to that of β‐actin, and the normalized IRS1 protein level at 0‐h time point was set as 1. IRS1 half‐life (t_1/2_) was determined from the exponential curve equation calculated using the one‐phase exponential decay model (prism 9 software) (*n* = 3). (C) Western blot analysis of phosphorylations of AKT (p‐AKT) and S6K (p‐S6K) in NL‐20 cell lines with stable overexpression of GFP, FBXL16, FBXL16ΔFbox or FBXL16ΔLRR that were stimulated with IGF1 or vehicle control after serum starvation (*n* = 3). (D) NL‐20 cell lines with stable overexpression of GFP, FBXL16, FBXL16ΔFbox or FBXL16ΔLRR were grown in soft agar. After 21 days, colonies were stained and quantified using imagej software. Values are normalized to the GFP control (arbitrarily set as 100) and representative wells are shown next to the bar graph (*n* = 4). In (B) and (D), values in graphs represent mean ± SEM and statistical significance were determined by two‐way ANOVA (*P < 0.05; **P < 0.01; ***P < 0.001).

Like other F‐Box proteins, FBXL16 contains an F‐Box domain and a substrate interacting domain: LRR region. We aimed to assess the functional importance of the F‐box and LRR domains of FBXL16 in regulating IGF1/IRS1/AKT signaling by generating NL‐20 cells with stable overexpression of FBXL16 mutants lacking either the F‐box motif (FBXL16ΔBox) or the LRR domain (FBXL16ΔLRR). First, by co‐immunoprecipitation and Western blot analysis, we determined that FBXL16 and FBXL16ΔFbox interacted with IRS1, whereas FBXL16ΔLRR showed no interaction (Fig. 6A), suggesting LRR domain is the motif for interaction with IRS1. Next, we examined the impact of these mutants on IRS1 stability. Interestingly, while overexpression of FBXL16 significantly increased the stability of IRS1, deletion of either the F‐box or LRR domain greatly diminished this effect (Fig. 6B). Consistent with these results, unlike FBXL16, overexpression of FBXL16ΔFbox or FBXL16ΔLRR had negligible effect on the phosphorylations of AKT and S6K (Fig. 6C) and had no significant effects on anchorage‐independent cell growth (Fig. 6D). These results demonstrate that both the F‐box and LRR domains are crucial for FBXL16 in promoting IRS1 stability, IRS1/AKT signaling, as well as cell growth.

## 4 Discussion

While *KRAS* activating mutations occur frequently in NSCLCs, there had been no targeted therapy available for treating this subtype of lung cancer until the recent approval of covalent inhibitors (e.g., sotorasib and adagrasib) specifically targeting KRASG12C for the treatment of patients with advanced NSCLC [5, 7, 8]. Unfortunately, patients either have intrinsic resistance to these drugs or inevitably develop resistance upon drug treatment [4, 27, 29, 30, 31, 32]. Thus, it is urgently needed to identify new therapeutic targets upon which drug(s) can be developed for preventing or overcoming KRAS mutant inhibitor resistance. In the current study, we have identified FBXL16 as a new potential therapeutic target for this need. First, we have found that FBXL16 protein expression is selectively upregulated in LUADs with *KRAS* mutations. Second, upon the depletion of FBXL16, LUAD‐*KRASG12C* cell lines (H23, H1792 and SW1573), which are intrinsically resistant to the KRASG12C inhibitor, became highly sensitive to sotorasib. Third, FBXL16 protein is nearly undetectable in non‐cancerous lung epithelial cell lines (NL‐20 and BEAS‐2B) and normal lung tissues adjacent to tumors (Fig. 1C), suggesting that targeting FBXL16 may cause little toxicity to normal cells/tissues. Fourth, we have noted that FBXL16 is essential in cell growth not only for LUADs with *KRASG12C* mutations but also for LUADs with other mutations, such as A549 with *KRASG12S* mutation and H441 with *KRASG12V* mutation. This is important in that besides G12C mutation, there are a variety of other *KRAS* mutations in LUADs and no personalized treatment is yet available for those patients. Taken together, our findings suggest that FBXL16 is an interesting therapeutic target for treating LUADs with activating *KRAS* mutations and would be particularly important for overcoming resistance to the drugs directly targeting activating KRAS mutants.

One interesting finding in this study is that FBXL16 protein expression is upregulated in LUADs, in particular those with KRAS mutations. We wondered whether FBXL16 expression level is upregulated by KRAS. However, we found that knockdown of KRAS did not have clear effect on FBXL16 protein levels (Fig. S6), indicating that FBXL16 is not directly regulated by KRAS signaling. The mechanisms governing *FBXL16* expression remain relatively elusive. A previous study suggested E2F1, a transcription factor with a known tumor‐promoting role [39], as an activator of the *FBXL16* gene promoter [40]. In triple negative breast cancer, *FBXL16* expression is downregulated by the p38/miR‐135b‐3p axis [14], suggesting that *FBXL16* can also be regulated at post‐transcriptional level. Notably, in mice, homozygous knockout of FBXL16 resulted in perinatal lethality [41], and FBXL16 was identified as a repressor of differentiation in embryonic stem cells along the cardiomyocyte lineage [11]. These findings suggest critical roles for *FBXL16* in normal development. However, the mechanisms regulating *FBXL16* expression and activity remain largely unknown. To date, only a single study demonstrated that FBXL16 transcript was induced in response to glucocorticoid in BEAS‐2B human airway epithelial cells [42].

Another important finding of our study is that IRS1 protein is also highly expressed selectively in LUADs with *KRAS* mutations, and its expression level is positively correlated with that of FBXL16. Interestingly, we have revealed that FBXL16 upregulates IRS1 protein stability and cellular level and thus promotes IRS1/AKT signaling, which we believe is a major mechanism by which FBXL16 promotes LUAD‐*KRAS* mutant cell growth and drug resistance. Our previous study revealed that FBXL16 upregulates MYC protein stability by antagonizing the activity of another F‐box protein FBW7 [13]. Given that F‐Box proteins βTRCP [35] and FBXW8 [36] were shown to promote IRS1 ubiquitination and proteosomal degradation, we tested whether FBXL16 upregulates IRS1 protein level through affecting βTRCP or FBXW8. However, silencing of either βTRCP or FBXW8 did not alter the effect of FBXL16 knockdown on IRS1 (Fig. S5), suggesting that FBXL16 regulates IRS1 independent of βTRCP and FBXW8. While we do not have compelling evidence showing that FBXL16 directly regulates IRS1 and the downstream PI3K/AKT/S6K signaling, our study demonstrates that FBXL16 interacts with IRS1 and plays important roles in upregulating IRS1 signaling and cell proliferation and migration.

Besides IRS1, IGF1 and IGF1 receptor (IGF1R) have been shown to be upregulated in LUADs and are implicated in tumor progression and therapeutic resistance [43], primarily through the activation of PI3K/AKT pathway. However, drugs targeting IGF1R and/or this signaling pathway have not been approved for treating LUADs due to the lack of significant patient benefit owing to low efficacy or high level of toxicity [44, 45]. Given that FBXL16 protein level is low or undetectable in normal lung tissues and is selectively upregulated in LUADs, in particular those with *KRAS* mutations, we can speculate that targeting FBXL16 protein might be a safer and more beneficial therapy. Thus, future work is warranted to validate the essential role of FBXL16 in promoting LUADs‐*KRAS g*rowth and drug resistance *in vivo* and to determine its druggability.

## 5 Conclusions

In summary, our study reveals that FBXL16 expression level is upregulated in LUADs with *KRAS* activating mutations. In this context, FBXL16 increases IRS1 protein stability, upregulates IRS1/AKT signaling and promotes LUAD cell growth, migration, and drug resistance. Our findings highlight the potential of FBXL16 as a therapeutic target for treating LUAD with *KRAS* activating mutations.

## Conflict of interest

The authors declare no conflict of interest.

## Author contributions

MM and WL conceived the study, designed, and performed experiments, analyzed, and interpreted data, wrote, and approved the manuscript.

## Supporting information


**Fig. S1.** IHC analysis using an anti‐FBXL16 antibody in comparison with a normal rabbit IgG control.
**Fig. S2.** Knockdown of FBXL16 decreased A549 cell proliferation.
**Fig. S3.** FBXL16 upregulates AKT phosphorylation.
**Fig. S4.** FBXL16 upregulates IRS1 protein stability.
**Fig. S5.** The effect of FBXL16 on IRS1 is independent on FBXW8 and β‐TRCP1/2.
**Fig. S6.** Knockdown of KRAS had little effect on FBXL16 protein level in LUAD cell lines.

## Data Availability

The data that support the findings of this study are included in Figs 1, 2, 3, 4, 5, 6 and Figs S1–S6 of this article and are available from the corresponding author upon reasonable request.
