# Supplementary material for: FBXL16 promotes cell growth and drug resistance in lung adenocarcinomas with KRAS mutation by stabilizing IRS1 and upregulating IRS1/AKT signaling
Source: Mol Oncol. 2024 Jan 17;18(3):762–77. doi: 10.1002/1878-0261.13554 (PMC10920083; doi:10.1002/1878-0261.13554)
Supplement: Supplementary file 1 — Fig. S1. IHC analysis using an anti‐FBXL16 antibody in comparison with a normal rabbit IgG control. Fig. S2. Knockdown of FBXL16 decreased A549 cell proliferation. Fig. S3. FBXL16 upregulates AKT phosphorylation. Fig. S4. FBXL16 upregulates IRS1 protein stability. Fig. S5. The effect of FBXL16 on IRS1 is independent on FBXW8 and β‐TRCP1/2. Fig. S6. Knockdown of KRAS had little effect on FBXL16 protein level in LUAD cell lines. [file MOL2-18-762-s001.pdf]

FBXL16 promotes cell growth and drug resistance in lung adenocarcinomas with *KRAS* mutation by stabilizing IRS1 and upregulating IRS1/AKT signaling

Marion Morel and Weiwen Long

Figure S1: IHC analysis using an anti-FBXL16 antibody in comparison with a normal rabbit IgG control.

Figure S2: Knockdown of FBXL16 decreased A549 cell proliferation.

Figure S3: FBXL16 upregulates AKT phosphorylation.

Figure S4: FBXL16 upregulates IRS1 protein stability.

Figure S5: The effect of FBXL16 on IRS1 is independent on FBXW8 and β-TRCP1/2.

Figure S6: Knockdown of KRAS had little effect on FBXL16 protein level in LUAD cell lines.

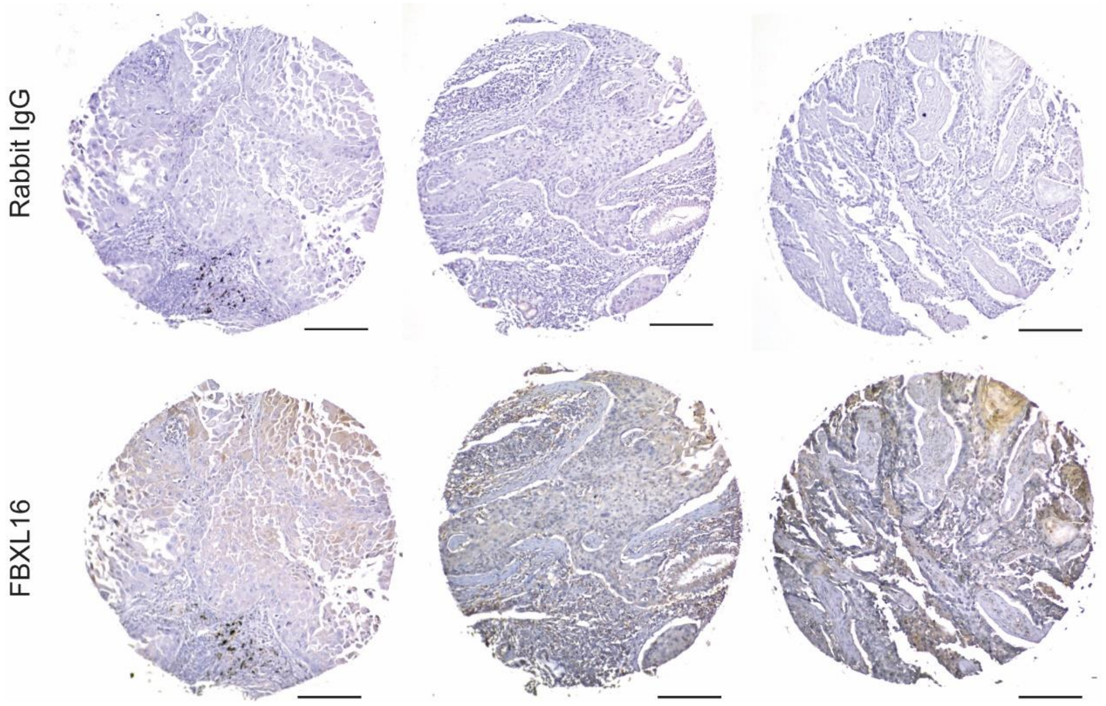

Figure S1: IHC analysis using an anti-FBXL16 antibody in comparison with a normal rabbit IgG control.

To evaluate the specificity of FBXL16 antibody for IHC analysis, TMAs were immunostained with either normal rabbit IgG (upper panels) or FBXL16 antibody (lower panels) at the same working concentration (1.7µg/mL) using the same conditions as Figures 1C and 1E. While the incubation with anti-FBXL16 antibody showed primarily cytoplasmic staining, incubation of the TMA with rabbit IgG showed negative staining (Scale bar: 250µm).

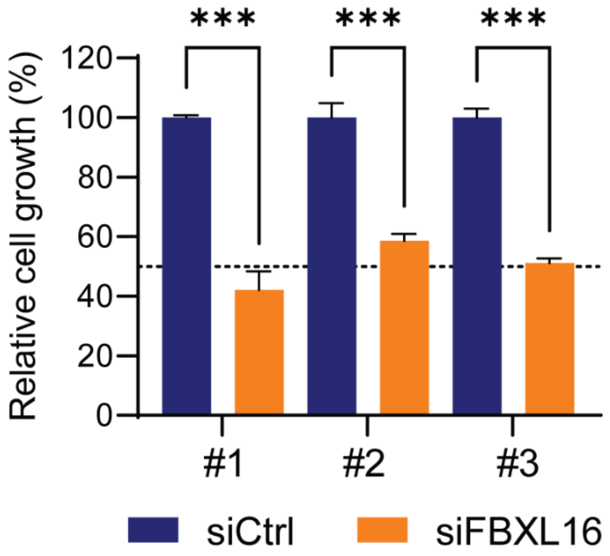

Figure S2: Knockdown of FBXL16 decreased A549 cell proliferation. A549 LUAD cells were transiently transfected individually with 3 different siRNA targeting FBXL16 (siFBXL16) (#1, #2 and #3) or each matched non-targeting control siRNA (siCtrl). 5 days post-transfection, cell growth was determined by dsDNA content measurement. Cell growth relative to each siCtrl is shown in bar graphs. # 1: Hs_FBXL16_8 FlexiTube siRNA (Qiagen SI04287276); #2: ON-TARGETplus Human FBXL16 siRNA (Horizon L-016797-00); #3: Hs_FBXL16_7 FlexiTube siRNA (Qiagen SI04277203).

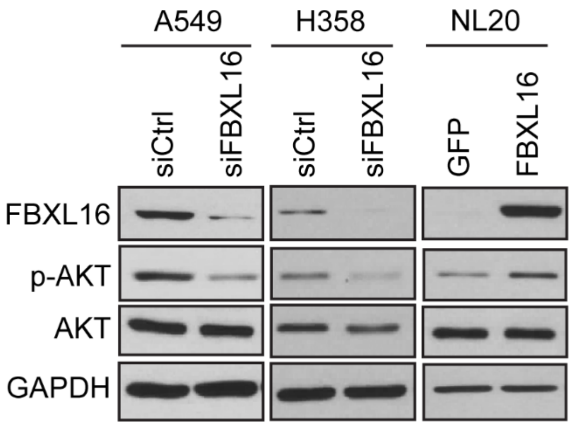

Figure S3: FBXL16 upregulates phosphorylation of AKT.

Western blot analysis of AKT phosphorylation (p-AKT) in A549 and H358 cells with transient knockdown of FBXL16 (siFBXL16 vs. siCtrl) or in NL20 cells with stable overexpression of FBXL16 or GFP control.

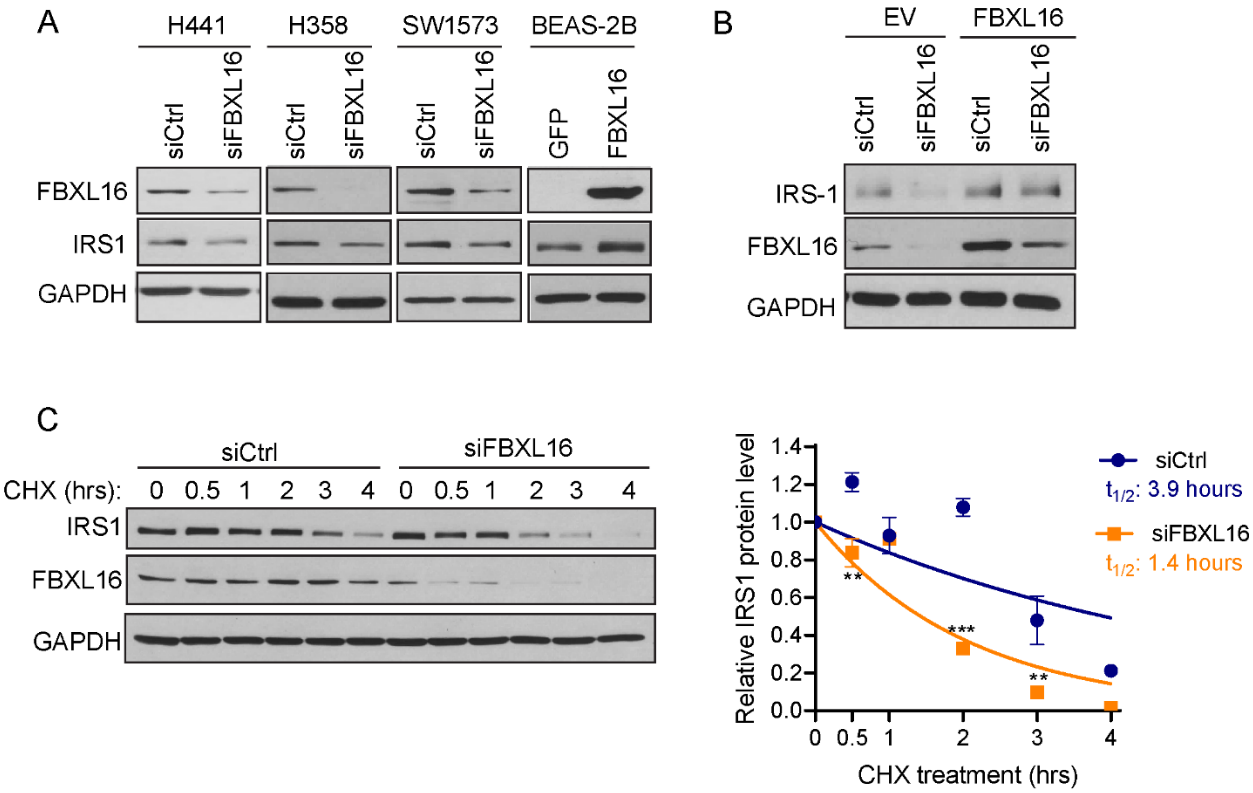

Figure S4: FBXL16 upregulates IRS1 protein stability.

(A) Western blot analysis after transient knockdown (H441, H358, SW1573 cells) or stable overexpression (BEAS-2B cells) of FBXL16. (B) Western blot analysis of IRS1 and FBXL16 in A549 cells with transfection of siCtrl or siFBXL16 together with transient overexpression of an empty vector (EV) control or FBXL16 cDNA. (C) H23 cells were transiently transfected with siCtrl or siFBXL16. 30 h post transfection, protein translation was inhibited with CHX (100µg/mL) for different time as indicated, followed by western blot analysis. IRS1 protein level at each time point was normalized to that of GAPDH, and the normalized IRS1 protein level at 0-hour time point was set as 1. IRS1 half-life ($t_{1/2}$) was determined from the exponential curve equation calculated using the one-phase exponential decay model (Prism 8.0 software). Values in graph represent mean ± SEM and statistical significance was determined by two-way ANOVA.

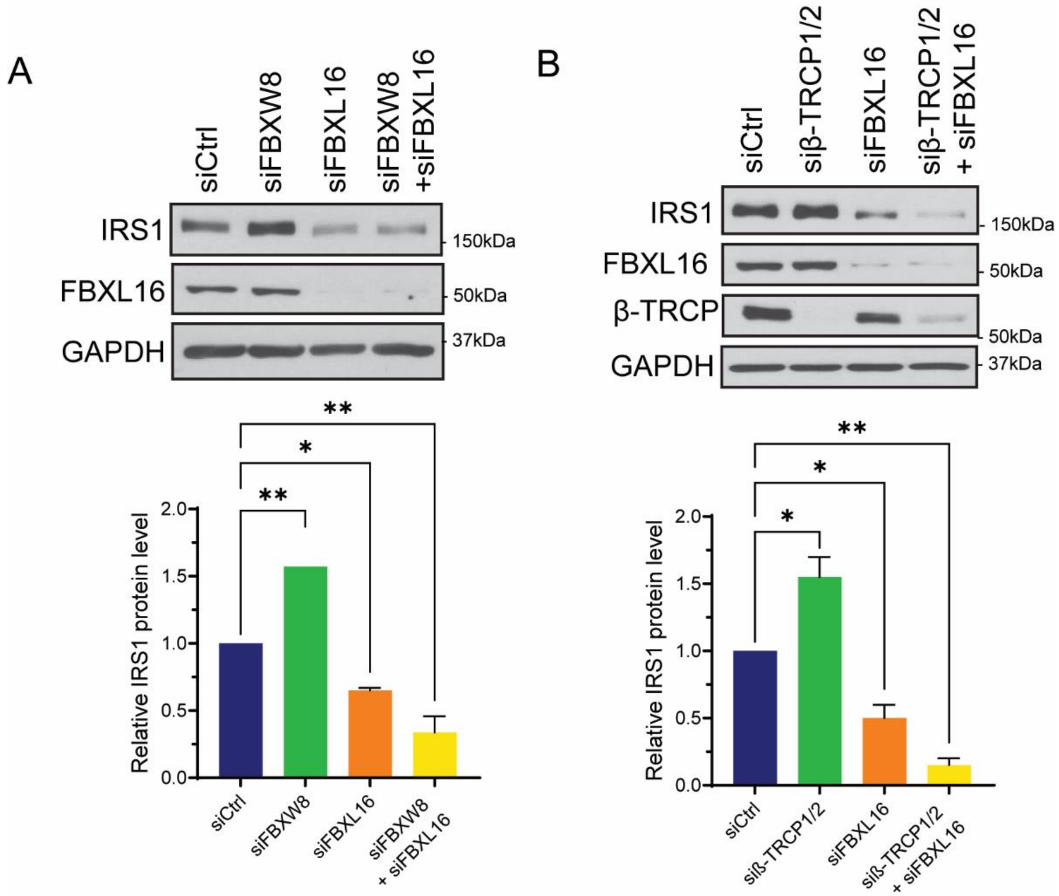

Figure S5: The effect of FBXL16 on IRS1 is independent on FBXW8 and β-TRCP1/2. (A) Western blot analysis and quantification of IRS1 protein level in A549 cells transiently transfected with negative control siRNAs (siCtrl), siRNA targeting FBXW8 (siFBXW8), siRNA targeting FBXL16 (siFBXL16) or both siFBXW8 and siFBXL16. (B) Western blot analysis and quantification of IRS1 protein level in A549 cells transiently transfected with negative control siRNAs (siCtrl), siRNA targeting β-TRCP1/2 (siβ-TRCP1/2), siRNA targeting FBXL16 (siFBXL16) or both siβ-TRCP1/2 and siFBXL16. Western blots are representative of 3 independent experiments. Values in graph bars represent mean ± SEM and statistical significance was determined by One-way ANOVA (*:p<0.05, **: p<0.01, ***: p<0.001).

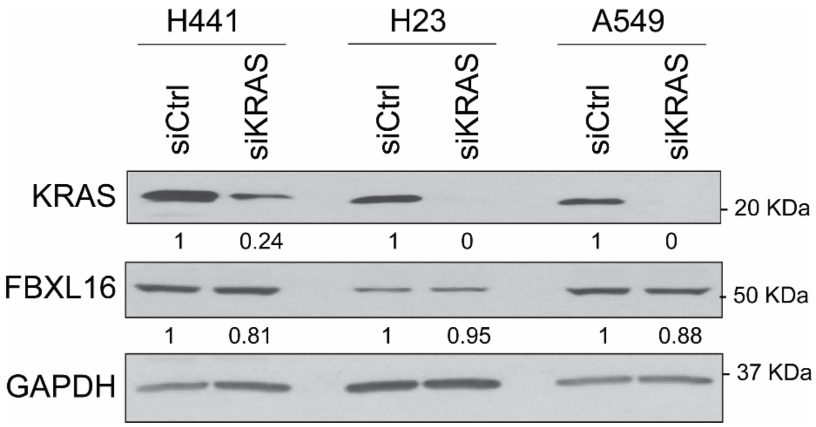

Figure S6: Knockdown of KRAS had little effect on FBXL16 protein level in LUAD cell lines.

Western blot analysis of FBXL16 and KRAS in H441, H23 and A549 cells transiently transfected with a negative control siRNA (siCtrl) or siRNA targeting KRAS (siKRAS). The number under each band indicates the band intensity of each protein relative to that of GAPDH.
